# Myocardial Metastases on 6-[^18^F] fluoro-L-DOPA PET/CT: A Retrospective Analysis of 116 Serotonin Producing Neuroendocrine Tumour Patients

**DOI:** 10.1371/journal.pone.0112278

**Published:** 2014-11-14

**Authors:** Walter Noordzij, André P. van Beek, René A. Tio, Anouk N. van der Horst-Schrivers, Elisabeth G. de Vries, Bram van Ginkel, Annemiek M. Walenkamp, Andor W. Glaudemans, Riemer H. Slart, Rudi A. Dierckx

**Affiliations:** 1 Department of Nuclear Medicine and Molecular Imaging, University of Groningen, University Medical Center Groningen, Groningen, The Netherlands; 2 Department of Endocrinology, University of Groningen, University Medical Center Groningen, Groningen, The Netherlands; 3 Department of Cardiology, University of Groningen, University Medical Center Groningen, Groningen, The Netherlands; 4 Department of Medical Oncology, University of Groningen, University Medical Center Groningen, Groningen, The Netherlands; 5 Faculty of Medicine, University of Groningen, University Medical Center Groningen, Groningen, The Netherlands; University of Bologna, Italy

## Abstract

**Purpose:**

This study evaluates the prevalence of cardiac metastases in patients with serotonin producing neuroendocrine tumours (NET), examined with ^18^F-FDOPA PET/CT, and the relationship of these metastases to the presence of carcinoid heart disease (CHD) based on echocardiography.

**Background:**

CHD occurs in patients with serotonin producing NET. The diagnostic method of choice remains echocardiography. The precise prevalence of cardiac metastases is unknown given the limitations of standard technologies. Nuclear medicine modalities have the potential to visualize metastases of NET.

**Methods:**

All patients who underwent ^18^F-FDOPA PET/CT because of serotonin producing NET between November 2009 and May 2012 were retrospectively analyzed. The presence of cardiac metastasis was defined as myocardial tracer accumulation higher than the surrounding physiological myocardial uptake. Laboratory tests and transthoracic echocardiography (TTE) results were digitally collected.

**Results:**

116 patients (62 male) underwent ^18^F-FDOPA PET/CT, mean age was 61±13 years. TTE was performed in 79 patients. Cardiac metastases were present in 15 patients, of which 10 patients also underwent TTE. One patient had both cardiac metastasis (only on ^18^F-FDOPA PET/CT) and echocardiographic signs of CHD. There were no differences in echocardiographic parameters for CHD between patients with and without cardiac metastases. TTE in none of the 79 patients showed cardiac metastases.

**Conclusion:**

The prevalence of cardiac metastases detected with ^18^F-FDOPA PET/CT in this study is 13%. ^18^F-FDOPA PET/CT can visualize cardiac metastases in serotonin producing NET patients. There appears to be no relationship between the presence of cardiac metastases and TTE parameters of CHD.

## Introduction

Neuroendocrine tumours (NET) are rare but well-defined neuroendocrine malignancies, with a low worldwide incidence; approximately 1–2 per 100,000 persons [1]. Nuclear medicine modalities and three-phase CT scans are the cornerstones in clinical staging of NET [1]. Indium-111 (^111^In) labelled pentetreotide scintigraphy (SRS, planar whole body scan with or without additional single photon emission computed tomography and CT (SPECT/CT)) and gallium-68 (^68^Ga) labelled (DOTA-Phe-Tyr)octreotide (DOTATOC) positron emission tomography (PET) are both used to visualize somatostatin receptor density. 6-[^18^F]fluoro-L-DOPA (^18^F-FDOPA) uptake in NET is analogous to the uptake of amine precursors in these tumours. The overall sensitivity of ^18^F-FDOPA PET/CT for the detection of (metastatic) NET lesions is higher than that of SRS [2]. All these nuclear medicine modalities have the potential to visualize tracer uptake in myocardial metastases in patient with NET [3], [4].

NET of the small bowel frequently produces serotonin which, in case of metastatic disease, may lead to carcinoid syndrome, consisting of diarrhoea, flushing and carcinoid heart disease (CHD). CHD, present in up to 60% of patients with carcinoid syndrome, is characterized by plaque-like endocardial deposits of fibrous tissue on the ventricular aspect of the tricuspid valve leaflets, the arterial side of the pulmonary valve cusps, but also in the right atrium, and right ventricle [5], [6]. This plaque formation and subsequent endocardial thickening eventually result in valve dysfunction [7]. Tricuspid valve involvement usually leads to regurgitation, whereas pulmonary valve involvement rather results into stenosis. Despite the indolent character of CHD, it is associated with a worse clinical outcome, especially when developing into heart failure [7].

Echocardiography is the modality of choice for diagnosing CHD [8], [9]. Characteristic findings consist of thickening of leaflets/cusps that become retracted and eventually immobile, leading to the combination of valvular regurgitation and stenosis. Myocardial metastases are sometimes considered as a feature of CHD. However, these metastases are not always detected with echocardiography, and do not necessarily lead to typical valve dysfunction [10], [11]. The prevalence of cardiac NET metastases is still not well established, partly due to lacking literature. Only few studies report on cardiac metastases, with a prevalence of 4% [12]. This is possibly an underestimation, partly due to low sensitivity of conventional imaging modalities.

The purpose of this retrospective study was to establish the prevalence of cardiac metastases in patients with serotonin producing NET, detected by ^18^F-FDOPA PET/CT, and its relationship with the presence of echocardiography based CHD.

## Patients and Methods

All patients who were referred to the Department of Nuclear Medicine and Molecular Imaging for an ^18^F-FDOPA PET/CT between November 2009 and May 2012, were retrospectively analyzed. Selected were patients with serotonin producing NET based on elevated urinary 5-hydroxyindole acetic acid (5-HIAA, upper reference limit 3.8 mmol/mol creatinine) and elevated serotonin in platelets (upper reference limit 5.4 nmol/10^9^ platelets). Serum creatinine level was used to determine the estimated glomerular filtration rate (eGFR). Presence of chronic kidney disease (CKD) was based on the Kidney Disease Outcomes Quality Initiative CKD classification [13].

Previous medical history was retrieved from the electronic patient chart, especially history of hypertension (defined as either systolic pressure higher than 140 mmHg, diastolic pressure higher than 90 mm Hg, or the use of anti-hypertensive medication), atrial fibrillation (AF) and myocardial infarction. Electrocardiograms (ECG) at the time point of the ^18^F-FDOPA PET/CT scan (±3 months) were collected from the electronic patient chart, when available. ECGs were re-evaluated regarding rhythm, heart rate, QRS axis, PQ interval, QRS duration, presence of bundle branch block, QTc interval and STT segments. Transthoracic echocardiography (TTE) findings at the same time point were collected from the local digital archive of the Department of Cardiology of our hospital. Patients were followed until May 2013: one year after the ^18^F-FDOPA PET/CT scan of the last included patient. Information on cardiac events (either myocardial infarction, or sudden cardiac death) or death during follow-up was retrieved from the electronic chart. This study was approved by the Institutional Ethics Review Board (name: ‘medisch ethische toetsingscommissie’ of the University Medical Center Groningen). According to the Dutch law, no additional informed consent was required. Patient information was anonymized and de-identified before data analysis.

### ^18^F-FDOPA PET/CT scanning

All patients fasted for at least 6 hours and were allowed to continue all medication. Whole-body (from top of the skull through midthigh) three-dimensional PET images were acquired 60 minutes after intravenous administration of a standard dose of 200 MBq ^18^F-FDOPA on a Biograph mCT (Siemens Medical Systems, Knoxville, TN, USA). ^18^F-FDOPA PET/CT scans were performed with a mean amount of 140±15.5 mg carbidopa premedication, and mean administered activity of 186±39.8 MBq ^18^F-FDOPA. Acquisition was performed in 7 bed positions of 2 minutes emission scan for patients 60–90 kg. Patient with body weight less than 60 kg and more than 90 kg body weight, were scanned with 1 minute and 3 minutes per bed position, respectively. Low dose transmission CT was used for attenuation correction. For the reduction of tracer decarboxylation and subsequent renal clearance, all patients received 2 mg/kg carbidopa (with a maximum of 150 mg) orally as pre-treatment, 1 hour before the ^18^F-FDOPA injection, to decrease peripheral uptake and to increase tracer accumulation in tumor cells. Low dose CT and ^18^F-FDOPA PET scans were automatically fused by use of three-dimensional fusion software (Siemens) with manual fine adjustments. All scans were interpreted by well trained nuclear medicine physicians as part of routine care. For this study, all scans were retrospectively analyzed for the presence of myocardial metastases.

Raw data were reconstructed through ultra high definition (Siemens) and guidelines based standardized algorithms [14], [15], for visual assessment and standardized uptake value (SUV) calculations, respectively. Focal left and/or right ventricle ^18^F-FDOPA uptake higher than the surrounding physiological myocardial uptake was considered abnormal, and thus suspicious for cardiac metastases.

### Transthoracic echocardiography

The following variables were measured according to standard recommendations in the M-mode transthoracic echocardiographic examination: left ventricular internal end-diastolic and end-systolic dimensions, inter-ventricular wall thickness and left ventricular posterior wall thickness at end-diastole. An eyeballing left ventricular ejection fraction (LVEF) >55% was considered to be normal, between 55% and 40% mildly disturbed, between 30% and 40% moderately disturbed, and <30% was considered to represent a severely impaired systolic function.

Serotonin induced cardiac involvement was defined as tricuspid or pulmonary valvular dysfunction (i.e. valve leaflet thickening, shortening, retraction, hypomobility, or incomplete coaptation) associated with regurgitation or stenosis [16], [17]. Pulmonary valve (PV) stenosis (peak gradient across the valve) was graded as mild (<25 mmHg), moderate (25 to 50 mmHg), or severe (>50 mmHg). Tricuspid valve (TV) stenosis (mean gradient across the valve) was graded as mild (0 to 5 mmHg), moderate (5 to 8 mmHg), or severe (>8 mmHg) [18]. Tricuspid annular plane systolic excursion (TAPSE) was measured by M-mode echocardiography at the RV free wall in the apical four-chamber view [19].

### Statistical analysis

Results are expressed as mean value ± standard deviation, or median (range) in the case of an abnormal distribution. The differences between patient categories were evaluated using unpaired Student’s *t*-tests (in case of normal distribution), independent sample test (Mann-Whitney U, in case of abnormal distribution), or Chi-square in case of categories. A *p* value <0.05 was considered significant. Survival was analyzed using log rank test. Statistical analysis was performed using the SPSS package version 20 (IBM Corp., Armonk, NY, USA).

## Results

### Patient characteristics

**Figure 1:**
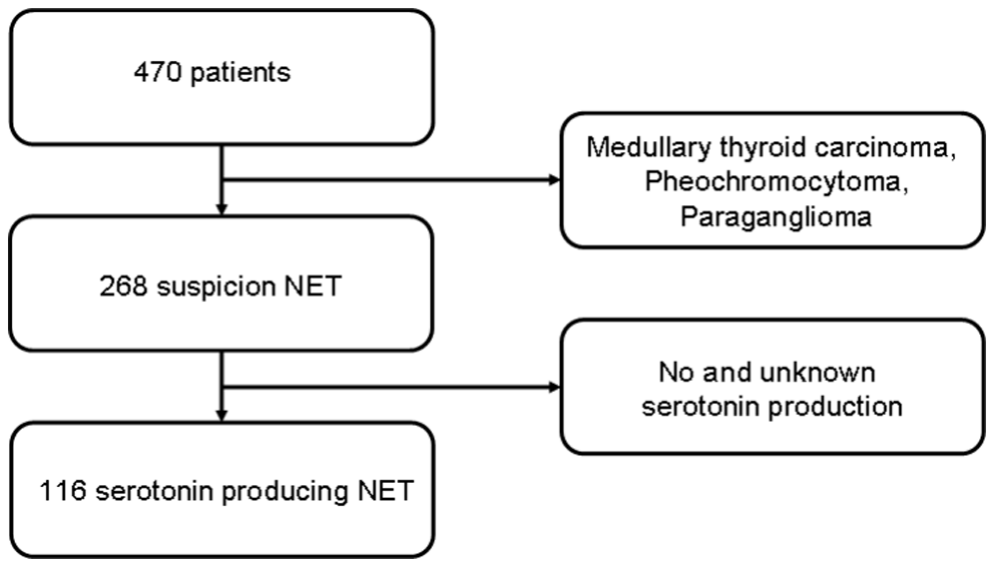
CONSORT diagram.

Patient selection is shown in Fig. 1.Of the 470 patients who underwent ^18^F-FDOPA PET/CT, those with medullary thyroid carcinoma, pheochromocytoma and paraganglioma (n = 202) were excluded. Of the remaining 268 patients with any form of NET, 116 patients had a serotonin producing NET. Of these patients 103 had a primary location in the small bowel, three a primary in lung, and in 10 patients the exact location of the primary tumour was unknown.

Characteristics of the 116 patients are presented in Table 1. There was a slight male predominance: 62 (53%) vs 54 female. None of the patients had recordings or complaints of ventricular dysrhythmia. During follow up (median 24, range 1–42 months), no cardiac event (either myocardial infarction, or sudden cardiac death) was reported. Eventually 30 patients (26%) died during follow-up. Metastatic disease was the main cause of death. Two patients died from non-disease related causes (one because of a cerebro-vascular accident, the other patient died due to pneumonia during the treatment of her co-existing non-Hodgkin’s lymphoma).

**Table 1:** Characteristics of all patients, and patients with and without abnormal cardiac ^18^F-FDOPA uptake.

	All patients		No cardiac metastases		Cardiac metastases		*p*-value
Parameter	N (%)	Mean ± SD, or median (range)	N (%)	Mean ± SD, or median (range)	N (%)	Mean ± SD, or median (range)	
Age (y)	116 (100)	64±9.3	101 (87)	64±8.5	15 (13)	67±8.1	NS
Male patients	62 (53)		55 (54)		7 (47)		NS
History of							
- Hypertension	48 (41)		39 (39)		9 (60)		NS
- Atrial fibrillation	3 (3)		2 (1)		0		NS
- Myocardial infarction	7 (6)		6 (6)		1 (7)		NS
Cardiac medication							
- β blocker	34 (29)		28 (28)		6 (40)		NS
- Angiotensin converting enzyme inhibitor	18 (16)		16 (16)		2 (13)		NS
- Diuretic	21 (18)		15 (15)		6 (40)		0.021
- Angiotensin II antagonist	9 (8)		8 (8)		1 (7)		NS
- Statin	28 (24)		25 (25)		3 (20)		NS
- Calcium channel blocker	12 (10)		9 (9)		3 (20)		NS
Laboratory tests	116 (100)		101 (87)		15 (13)		
Serum							
- Creatinine (µmol/L)	114 (98)	84±29	99 (98)	84±30	15 (100)	80±6	NS
- Estimated glomerular filtration rate (mL/min/1.73 m^2^)	114 (98)	77±20	99 (98)	78±21	15 (100)	72±18	NS
- Chromogranin A (µg/L)	116 (100)	185 (29.0–44.6×10^3^)	101 (100)	168 (29.0–44.6×10^3^)	15 (100)	320 (48.0–19.1×10^3^)	NS
- Platelet serotonin (nmol/10^9^)	116 (100)	18 (3.8–58)	101 (100)	17 (3.8–48)	15 (100)	20 (8.2–58)	NS
Urine							
- Serotonin (µmol/mol creatinine)	98 (84)	69 (16–1.2×10^3^)	89 (88)	67 (16–1.2×10^3^)	9 (60)	96 (36–4.3×10^2^)	NS
- 5-Hydroxyindole acetic acid (mmol/mol creatinine)	110 (95)	8.5 (1.8–3.9×10^2^)	96 (95)	7.3 (1.8–2.3×10^2^)	14 (93)	11 (3.2–3.9×10^2^)	NS
Electrocardiography	60 (52)		51 (50)		9 (60)		
- Heart rate (bpm)	60 (100)	68±17	51 (100)	68±17	9 (100)	67±24	NS
- Sinus rhythm	58 (97)		50 (98)		8 (89)		NS
- Atrial fibrillation (n)	2 (3)		1 (1)		1 (11)		NS
- PQ duration (ms)	58 (97)	158±23	50 (98)	157±22.6	8 (89)	162±23.2	NS
- QRS duration (ms)	60 (100)	90±15	51 (100)	90±15	9 (100)	93±10	NS
- QTc duration (ms)	60 (100)	416±28	51 (100)	416±29.0	9 (100)	420±25.0	NS

In 114 patients (98%), eGFR as a marker for kidney function was determined. Mean eGFR was within normal ranges. In 22 patients, eGFR was moderately decreased (values 30–60 mL/min*1.73 m^2^), however, none of the patients had severe impaired kidney function, defined as eGFR <30 mL/min*1.73 m^2^.

An ECG in the period of the ^18^F-FDOPA PET/CT was available in 60 patients (52%). The majority of patients had sinus rhythm at the time of the ECG. In two patients (2%) AF was recorded. One of these patients was newly diagnosed with AF, whereas also one patient with previous reported AF was in sinus rhythm at the time of ECG registration.

### ^18^F-FDOPA PET/CT findings

**Figure 2:**
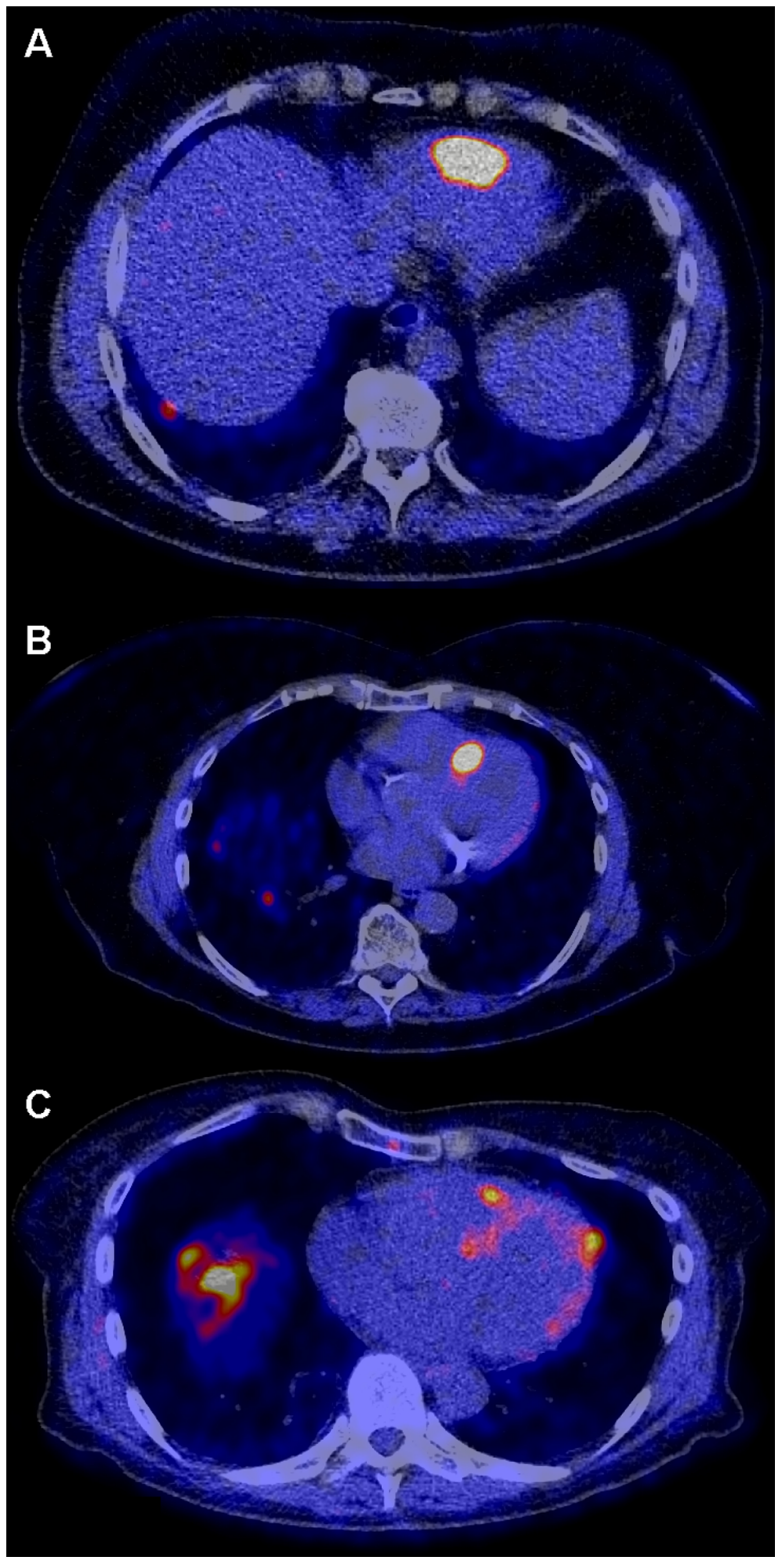
Three fusion images of ^18^F-FDOPA PET/CT scan in three different patients, indicating cardiac metastases. A: Intense tracer accumulation in the apex of the right ventricle wall. B: Intense uptake in the inter-ventricular septum. C: One lesion in the apex of both the left and right ventricle, with physiological uptake in the rest of the myocardium. The more diffuse and intense uptake projected in the basal part of the right lung is actually located in large liver metastases.

Table 2 shows the results of the ^18^F-FDOPA PET/CT scans. Fifteen patients (13%) showed abnormal tracer uptake (focal myocardial uptake higher than the surrounding physiological myocardial uptake), suspected of being a result of myocardial metastases of the NET. Of these 15 patients, one patient had a serotonin producing NET originating from the lung. All others had serotonin producing NET originating from the small bowel. Three patients had manifestation of one cardiac metastasis in the left ventricle only, six patients only one metastasis in the right ventricle free wall, and six patients showed lesions in both left and right ventricle walls (Fig. 2).

**Table 2:** 6-[fluoride-18]fluoro-L-DOPA (^18^F-FDOPA PET/CT) results.

	All patients		No cardiac metastases		Cardiac metastases		*p*-value
Parameter	N (%)	Mean ± SD	N (%)	Mean ± SD	N (%)	Mean ± SD	
^18^F-FDOPA PET/CT scan	116 (100)		101 (87)		15 (13)		
SUV_max_ right ventricle	116 (100)	2.1±1.6	101 (87)	1.9±0.41	15 (13)	3.9±4.4	<0.001
SUV_mean_ right ventricle	116 (100)	1.4±0.37	101 (87)	1.3±0.32	15 (13)	1.6±0.65	0.015
SUV_max_ left ventricle	116 (100)	2.9±1.6	101 (87)	2.8±1.3	15 (13)	3.7±2.8	0.029
SUV_mean_ left ventricle	116 (100)	1.7±0.36	101 (87)	1.7±0.35	15 (13)	1.7±0.47	NS

SUV denotes Standardized Uptake Value.

Of these 15 patients, 4 (27%) died during follow-up after the diagnosis of cardiac metastasis (median 5, range 0–22 months). This was not significantly different from 26 (26%) patients who died with normal cardiac tracer distribution (median 17, range 2–37 months).

### Echocardiography

Echocardiography was performed in 79 patients (68%, Table 3). The median time between the ^18^F-FDOPA PET/CT and echocardiography was 0.80 (range 0.20–1.4) months. Mean LV wall thickness and atrial diameter measurements were within normal ranges. In 10 patients inter-ventricular septum thickness was more than 11 mm, whereas posterior wall was thicker than 11 mm in three patients.

**Table 3:** Echocardiography results of all patients, and patients with and without abnormal cardiac tracer uptake.

	All patients		No cardiac metastases		Cardiac metastases		*p*-value
Parameter	N (%)	Mean ± SD, or median (range)	N (%)	Mean ± SD, or median (range)	N (%)	Mean ± SD, or median (range)	
Heart rate (bpm)	72 (91)	71±18	64 (91)	72±17	8 (89)	68±27	NS
Inter-ventricular septal thickness (mm)	63 (88)	9.4±1.8	54 (77)	9.2±1.7	9 (100)	11±2.1	0.037
Left ventricle posterior wall thickness (mm)	63 (88)	8.5±1.7	54 (77)	8.4±1.6	9 (100)	9.3±2.0	NS
Left ventricle end diastolic volume (mL)	38 (48)	96.8 (41.3–214)	34 (49)	95.2 (41.3–214)	4 (44)	116 (87.2–160)	NS
Left ventricle end systolic volume (mL)	40 (51)	30 (18–95)	36 (51)	30 (18–95)	4 (44)	58 (20–90)	NS
Left ventricle diastolic internal diameter (mm)	63 (88)	46 (34–76)	54 (77)	46 (34–76)	9 (100)	50 (36–58)	NS
Left ventricle systolic internal diameter (mm)	64 (91)	28 (22–46)	55 (79)	28 (22–46)	9 (100)	34 (23–44)	NS
Left ventricle ejection fraction (%)	62 (78)	60±9.8	54 (77)	61±8.6	8 (89)	52±14	0.010
Left atrium diameter (mm)	57 (72)	35±5.6	50 (71)	35±5.6	7 (78)	35±6.3	NS
Right atrium length (mm)	55 (70)	52±5.3	49 (70)	52±5.3	6 (67)	55±5.0	NS
Right atrium wide (mm)	50 (63)	39±6.6	45 (64)	40±6.5	5 (56)	39±8.7	NS
Left atrium length (mm)	53 (67)	54±8.1	47 (67)	54±8.4	6 (67)	55±4.6	NS
Left atrium wide (mm)	53 (67)	40±6.0	47 (67)	39±5.9	6 (67)	45±4.8	0.019
Pulmonary valve peak flow (mmHg)	45 (57)	7.7±6.2	37 (53)	7.9±5.9	8 (89)	6.9±7.7	NS
- Mild pulmonary valve stenosis (<25 mmHg)	45 (100)		37 (100)		8 (100)		NS
Tricuspid valve peak gradient (mmHg)	11 (14)	3.3±3.1	10 (14)	3.4±3.3	1 (11)	2.2	NS
- Mild tricuspid valve stenosis (<5 mmHg)	7 (64)		6 (60)		1 (100)		NS
- Moderate tricuspid valve stenosis (5–8 mmHg)	4 (36)		4 (40)		0		NS
Tricuspid valve regurgitation peak flow (mmHg)	37 (47)	24±7.2	29 (41)	23±7.1	8 (89)	28±6.7	NS
TAPSE (mm)	63 (80)	24±4.4	56 (80)	24±4.3	7 (78)	26±4.7	NS

TAPSE denotes tricuspid annular plane systolic excursion.

**Figure 3:**
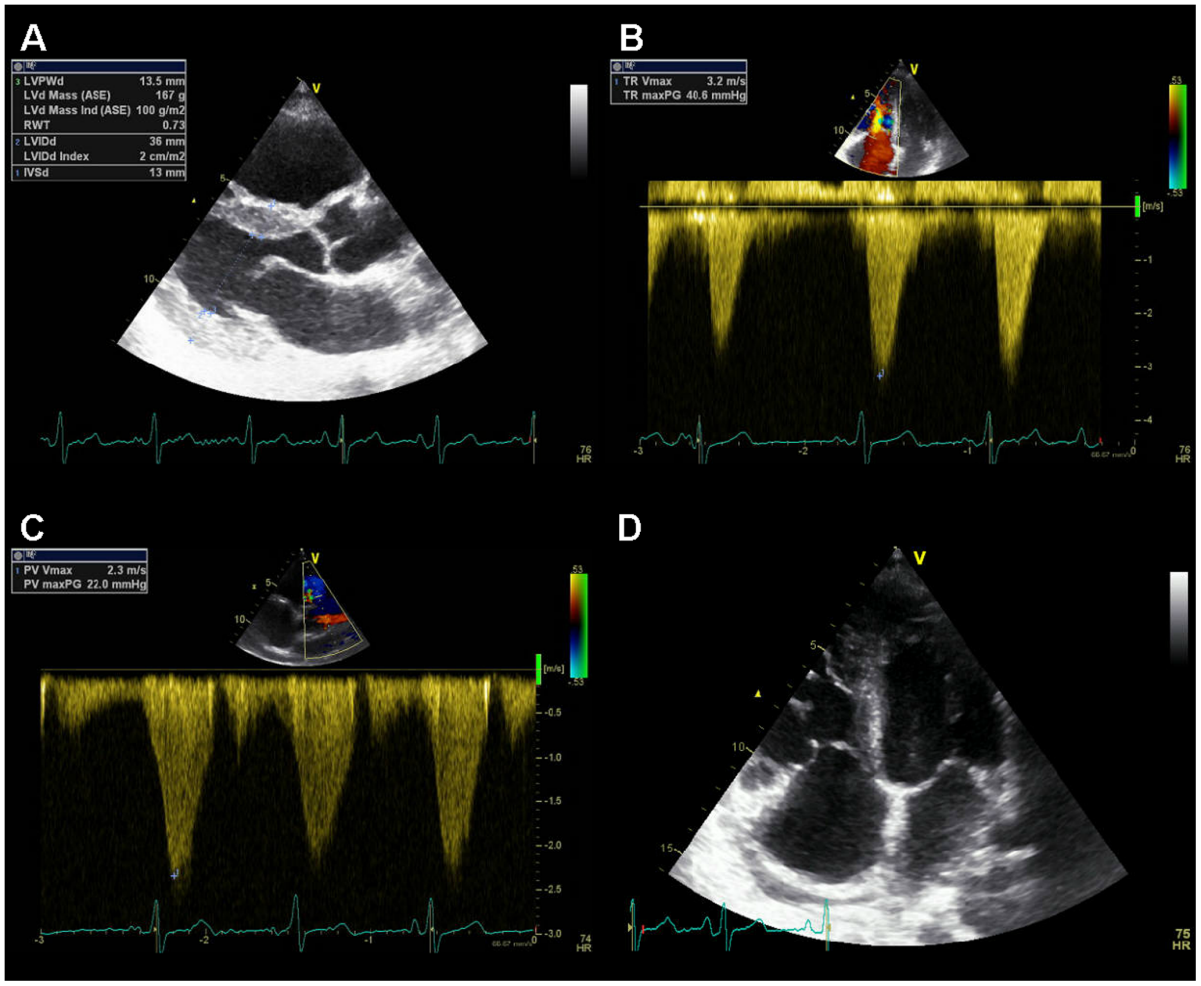
Example of echocardiographic characteristics of a patient with carcinoid heart disease. A: Parasternal long axis view showing thickened LV walls (inter-ventricular septum 13 mm, posterior wall 13 mm). B: Continuous wave Doppler of the tricuspid valve jet. The peak velocity (41 mm Hg) indicates severe tricuspid insufficiency. C: Continuous wave Doppler of the pulmonary valve. The peak gradient (22 mmHg) indicates mild pulmonary valve stenosis. D: 2D four chamber image. On the right in the image normal function of the mitral valve. On the left in the image retraction and immobilisation of the tricuspid valve. The leaflets of the tricuspid valves are also hyperechogenic than normal.

**Figure 4:**
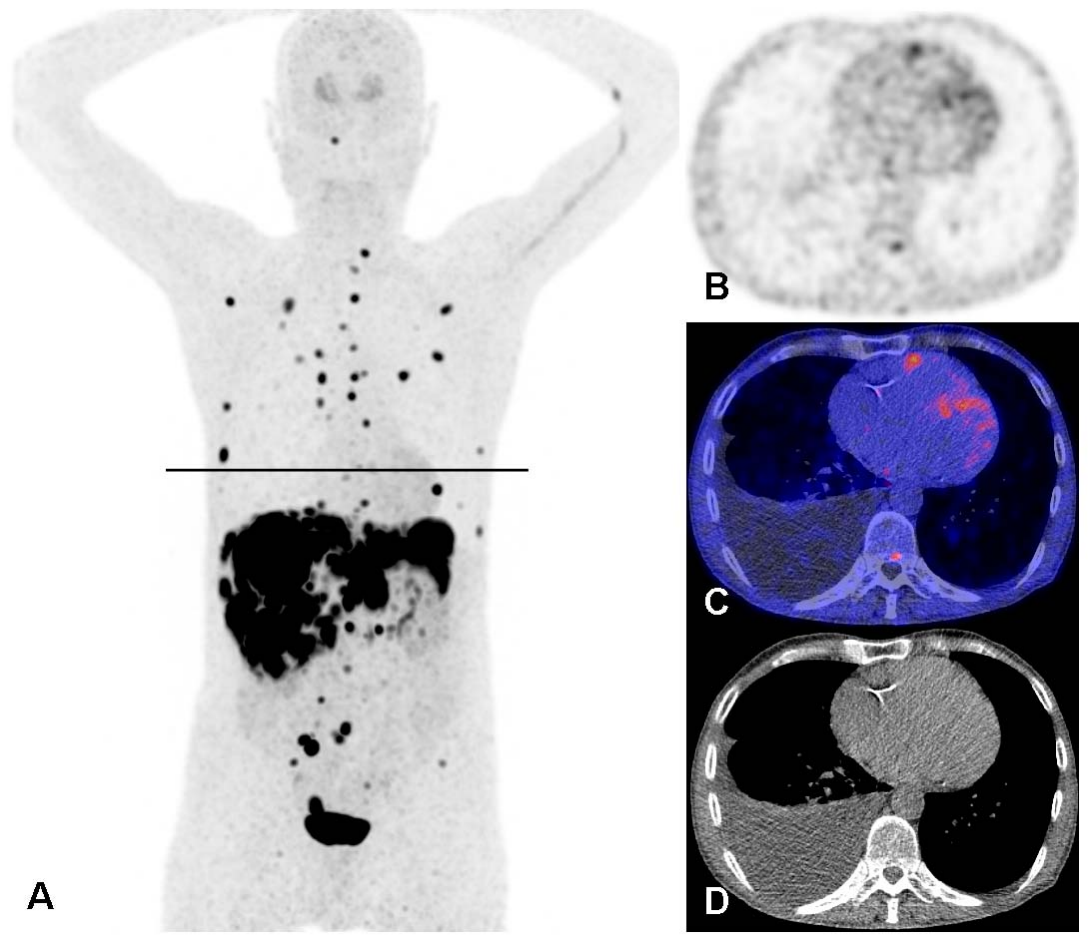
^18^F-FDOPA PET/CT scan of the patient illustrated in **Fig 2**. A: Maximum intensity projection showing multiple metastases of the carcinoid tumour in the mediastinum, thoracic wall, liver, mesentery and skeleton. Physiological uptake in the striatum. Administration artefact in the left elbow and vein in the left upper arm. The black line is the slice position of panels B, C, and D. PET alone (B), fused PET/CT (C) and CT alone (D) images on the slice position indicated in panel A. Physiological uptake in the myocardium with a focus of increased uptake in the right ventricle wall. Also small focal uptake in the posterior part of a thoracic vertebral body, right sided pleural effusion and atherosclerosis of the right coronary artery.

Of these 79 patients, nine patients had abnormal cardiac ^18^F-FDOPA uptake (Table 3). In six patients with abnormal cardiac uptake, no echocardiography was performed. All of these 15 patients had metastatic disease, based upon the ^18^F-FDOPA PET/CT. Of the echocardiographic parameters, septum thickness, LVEF, left atrium (wide) measurement and mean peak flow across the aortic valve appeared to be significantly different in the patients with abnormal cardiac tracer distribution. Only one of the nine patients with abnormal ^18^F-FDOPA uptake had echocardiographic signs of carcinoid heart disease: hypertrophic left ventricle (both septum and posterior wall thickness 13 mm), estimated ejection fraction of 30%, severe tricuspid valve (peak gradient 41 mmHg) and pulmonary valve insufficiency, and a mild pulmonary stenosis (peak gradient 22 mmHg, Figs. 3 and 4). In none of the patients with abnormal ^18^F-FDOPA uptake in the LV wall who underwent echocardiography a patent foramen ovale could be retrieved on the saved images.

**Figure 5:**
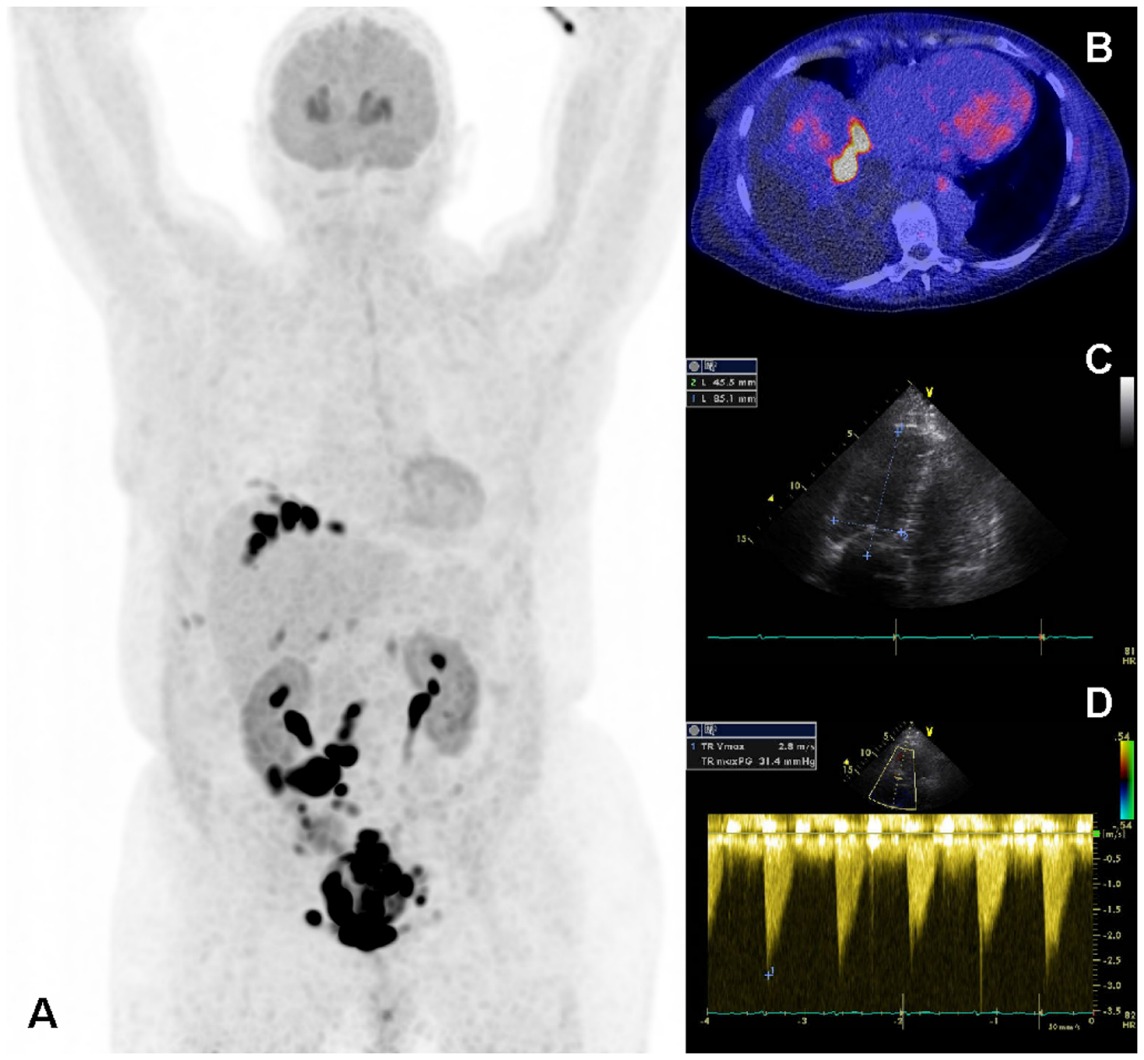
Maximum intensity projection (A) of an ^18^F-FDOPA PET/CT scan in a patient with echocardiographic signs of carcinoid heart disease, but without myocardial metastases on the ^18^F-FDOPA PET/CT scan. B: Fused PET/CT image showing only physiological tracer distribution in the myocardium. The intense uptake in liver is due to liver metastases. Ascites surrounding the right liver half. C: Measurements of the right ventricle (85 mm long, 46 mm wide). D: Continuous wave Doppler of the tricuspid valve (peak gradient 31 mmHg), indicating tricuspid insufficiency.

Seven patients with echocardiographic parameters of CHD showed no signs of myocardial metastases on the ^18^F-FDOPA PET/CT. However, the right ventricle diameter in these patients appeared to be relatively wide (Fig. 5). In two of these patients CHD was based on relatively high maximum peak gradient of TV regurgitation (31 and 37 mm Hg) and peak gradient across the PV (22 and 16 mmHg). In this small group of CHD patients urine level of 5-HIAA was higher than those without echo-based CHD: median 153 (range 4.30–385) vs 5.70 (range 1.70–160) mmol/mol creatinine, *p* = 0.001).

## Discussion

This study showed cardiac metastases in 15 out of 116 patients (13%). The presence of cardiac metastases was not related to the occurrence of CHD.

There is slowly growing awareness that cardiac metastases occur rather frequently. However, the early detection of these metastases, especially with echocardiography, remains a challenge. The main limitation is that lesions smaller than 10 mm are non-detectable [20]. The conventional nuclear medicine modality ^111^In-SRS has the same limitation, especially in whole body imaging. The addition of SPECT/CT of the thorax contributes to a better spatial resolution, with more precise anatomic localization of abnormal tracer accumulation. The introduction of newer imaging modalities with PET tracers ^68^Ga-DOTATOC and ^18^F-FDOPA provide an even better spatial resolution (up to 4 mm) and overall more sensitive detection of metastases of NET [4]. This is the largest cohort of patients referred for ^18^F-FDOPA PET/CT scans, showing that cardiac metastases of NET are more prevalent than previously assumed.

In a review of the reported literature thus far in 2010, a total of 45 patients with cardiac metastases based on imaging, surgery and autopsy findings were retrospectively analyzed [4]. In 21 cases, the presence of cardiac metastasis was based on echocardiographic findings, whereas nuclear medicine modalities were positive in 10 patients. PET scans visualized cardiac metastases in three out of those 10 cases. In all of these cases, the presence of cardiac metastases could be verified with other imaging modalities: echocardiography and MRI. This indicates that both PET tracers are accurately able to visualize cardiac metastases. A recent report on rare metastases of NET further confirms this statement [21]. In this study a total of 4,210 ^68^Ga-somatostatin-receptor PET/CT scans performed in a 5-year period were retrospectively analyzed. Cardiac metastases appeared to be rare: in merely 29 of all cases. Both these reports also included non-serotonin producing and non-metastatic NET. Therefore, the prevalence of cardiac metastases in these cohorts is less than 1%. However, this is even less than the low prevalence of 4%, as mentioned previously, and probably a result of selection bias [12]. On the other hand, the most recent study on the presence of cardiac metastases in patients with NET of the small bowel reported that cardiac metastases detected by ^68^Ga-DOTATOC PET/CT were present in four out of 92 patients with ileal NET [22]. These four patients showed in total seven cardiac metastases, supporting the previously acclaimed prevalence of approximately 4%. As in our study, all patients with myocardial metastases had high tumour burden. Although cardiac metastases are usually detected in patients with widespread metastatic disease, the myocardium can occasionally be the only location of NET metastases [4]. Our study consists of the largest cohort of patients referred for ^18^F-FDOPA PET/CT scans. We clearly add the novel information that cardiac metastases of NET occur more frequently than previously assumed, and that ^18^F-FDOPA PET/CT appears to be more sensitive in detecting these metastases than other imaging modalities reported so far.

We showed no relationship between the presence of cardiac metastases and echocardiographic parameters for CHD. This suggests that myocardial metastases and typical CHD are two different entities, which do not seem to affect each other. We could not identify a patent foramen ovale in patients with cardiac metastases, which may support the idea that the presence of cardiac metastases is independent of flow. Lower eyeballing LVEF and wider left atrium measurements were considered to be coincidental findings due to the small group of patients. Urinary excretion of 5-HIAA was higher in patients with echocardiographic signs of CHD, but not in patients with cardiac metastases. Although the duration of the disease was not investigated in this retrospective study, this finding implies that CHD patients probably had long standing disease. Other laboratory tests, as well as ECG findings did not differ. This finding is concordant with the progression of CHD [23].

## Conclusions

The prevalence of cardiac metastases found on ^18^F-FDOPA PET/CT is higher that previously assumed. Cardiac metastases were not related with typical echocardiographic features of CHD, suggesting that these findings are two different entities within the same disease. Of importance, the clinical consequences of this higher incidence of cardiac metastases found on ^18^F-FDOPA PET/CT may not be of high importance, due to the indolent character of the disease, leading to a comparable survival in patients without cardiac metastases. A large prospective study of serotonin producing NET patients with cardiac metastases should be performed to further establish the clinical role of ^18^F-FDOPA PET/CT for this indication.
